# Microinvasive breast cancer and the role of sentinel lymph node biopsy

**DOI:** 10.1038/s41598-022-16521-8

**Published:** 2022-07-20

**Authors:** Sean M. Hacking, Kara-Lynne Leonard, Dongling Wu, Mara Banks, Theresa Graves, Lijuan Wang, Evgeny Yakirevich, Yihong Wang

**Affiliations:** 1 Department of Pathology and Laboratory Medicine, Rhode Island Hospital and Lifespan Medical Center, Warren Alpert Medical School of Brown University, 593 Eddy Street, Providence, RI 02903 USA; 2 Department of Radiation Oncology, Rhode Island Hospital and Lifespan Medical Center, Warren Alpert Medical School of Brown University, Providence, USA; 3 Department of Pathology and Laboratory Medicine, Donald and Barbara Zucker School of Medicine at Hofstra/Northwell, New York, USA; 4 Department of Surgery, Rhode Island Hospital and Lifespan Medical Center, Warren Alpert Medical School of Brown University, Providence, USA

**Keywords:** Risk factors, Surgical oncology, Breast cancer

## Abstract

Whether sentinel lymph node biopsy (SLNB) should be performed in patients with microinvasive breast cancer (MIBC) has been a matter of debate over the last decade. MIBC has a favorable prognosis and while metastasis to the axilla is rare, it can impact treatment recommendations. In this study we evaluated clinical and histological features in both MIBC and background DCIS including ER, PR, and HER-2, number of foci of MIBC, the extent of the DCIS, nuclear grade, presence of comedo necrosis, as well as surgical procedures, adjuvant treatment and follow up to identify variables which predict disease free survival (DFS), as well as the factors which influence clinical decision making. Our study included 72 MIBC patients with a mean patient follow-up time of 55 months. Three patients with MIBC had recurrence, and two deceased, leaving five patients in total with poor long-term outcomes and a DFS rate of 93.1%. Performing mastectomy, high nuclear grade, and negativity for ER and HER-2 were found to be associated with the use of SLNB, although none of these variables were found to be associated with DFS. One positive lymph node case was discovered following SLNB in our study. This suggests the use of SLNB may provide diagnostic information to some patients, although these are the anomalies. When comparing patients who had undergone SLNB to those which had not there was no difference in DFS. Certainly, the use of SLNB in MIBC is quite the conundrum. It is important to acknowledge that surgical complications have been reported, and traditional metrics used for risk assessment in invasive breast cancer may not hold true in the setting of microinvasion.

## Introduction

Microinvasive breast cancer (MIBC) is defined as invasion of less than 1 mm into adjacent stroma^1^. Prior to this there was discrepant reporting of MIBC, with different definitions of microinvasion^1–6^, resulting in significant controversy. MIBC arises in the setting of DCIS and generally, patients diagnosed with DCIS have a normal life expectancy and a long-term survival of around 98% after 10 years^7,8^. Like DCIS, MIBC has been reported to be associated with good overall clinical outcomes. For example, Kwon et al.^9^ showed the 5-year recurrence free survival to be 97.2%, although after 10 years of follow up Parikh et al.^10^ showed a 10-year rate of recurrence free survival to be 90.7%. Most recently, based on the records review of 525,395 women, Sopik et al.^11^ demonstrated 20-year breast cancer-specific mortality to be 3.8% for pure DCIS, and 6.9% for MIBC, with an adjusted hazard ratio for death associated with MIBC when compared to pure DCIS to be 2.00 (95% CI 1.76–2.26; *p* < 0.0001).

Compared to DCIS, MIBC is seen in association with high nuclear grades, necrosis, human epidermal growth factor receptor 2 (HER-2) positivity and a high Ki-67 positivity index, whereas the rates of estrogen receptor (ER) and progesterone receptor (PR) positivity are lower in patients with microinvasive carcinoma arising the background of extensive DCIS^12^. For the purposes of treatment decision making, validating the reproducibility for different methods of risk stratification in MIBC will be important. The role of sentinel lymph node biopsy (SLNB) in MIBC is currently not well defined, while the rate of axillary metastases has been observed to be very low (0–11%)^10,13^. In a large study of 2609 patients with MIBC who underwent SLNB, only 76 (2.9%) patients were found to have sentinel lymph node metastases^14^.

Therapeutic approaches can result in overtreatment of some patients with breast cancer. The Marmot Report published in 2012 acknowledge the negative effects of overtreatment to women’s health^15^. There are many considerations for surgical interventions: poor cosmesis after surgery, chronic pain due to sentinel lymph node biopsy procedure in the axilla, and the possibility of no long-term outcome difference following procedure^13^. ER, PR, and HER-2 status have been extensively studied in invasive breast cancer, while less data is available regarding ER, PR and HER-2 in MIBC^6,16,17^. Although highly prevalent, there is little direct evidence that hormonal status is to be associated with improved long term outcomes in MIBC^18^. It is generally accepted that hormone receptor (HR) positive patients receive benefit from adjuvant endocrine therapy, however adjuvant chemotherapy in MIBC has been found to only improve the outcomes of ER(−)/PR(−) patients which did not overexpress Ki-67^19^.

Performing a risk assessment is important for guiding treatments and for MIBC there is limited information. Most research has not analyzed all pathology parameters together with clinical follow up. The principle aim of this study is to provide a comprehensive evaluation of MIBC and associated DCIS in relation to salient clinical and pathological variables including ER, PR, and HER-2 status, number of foci of MIBC, the size extent of the background DCIS and nuclear grade, presence of comedo-necrosis, as well as surgical procedures, adjuvant treatment and clinical follow-up to identify variables which predict disease free survival and influence clinical decision making.

## Materials and methods

### Case selection

Upon Lifespan Health System Institutional review board (IRB) approval (Lifespan IRB: 751551-10), a retrospective natural language search of the pathology database (Cerner CoPath) for patients over the age 18 years was performed from July 2010 to May 2020. Cases with a diagnosis of “microinvasive breast cancer” were retrieved, while definitively invasive carcinomas (> 1 mm) were excluded. Mammography was the single most common approach to breast cancer detection in this cohort of patients and all current AJCC protocols were followed according to the most current recommendations for the diagnosis of microinvasion (< 1 mm). Follow up data for death and recurrence was provided by the cancer registry at the Lifespan Health System. All methods were carried out in accordance with relevant guidelines and regulations and informed consent was not required by the Lifespan Health System Institutional review board secondary to the retrospective nature of this study.

### Pathology examination

**Figure 1:**
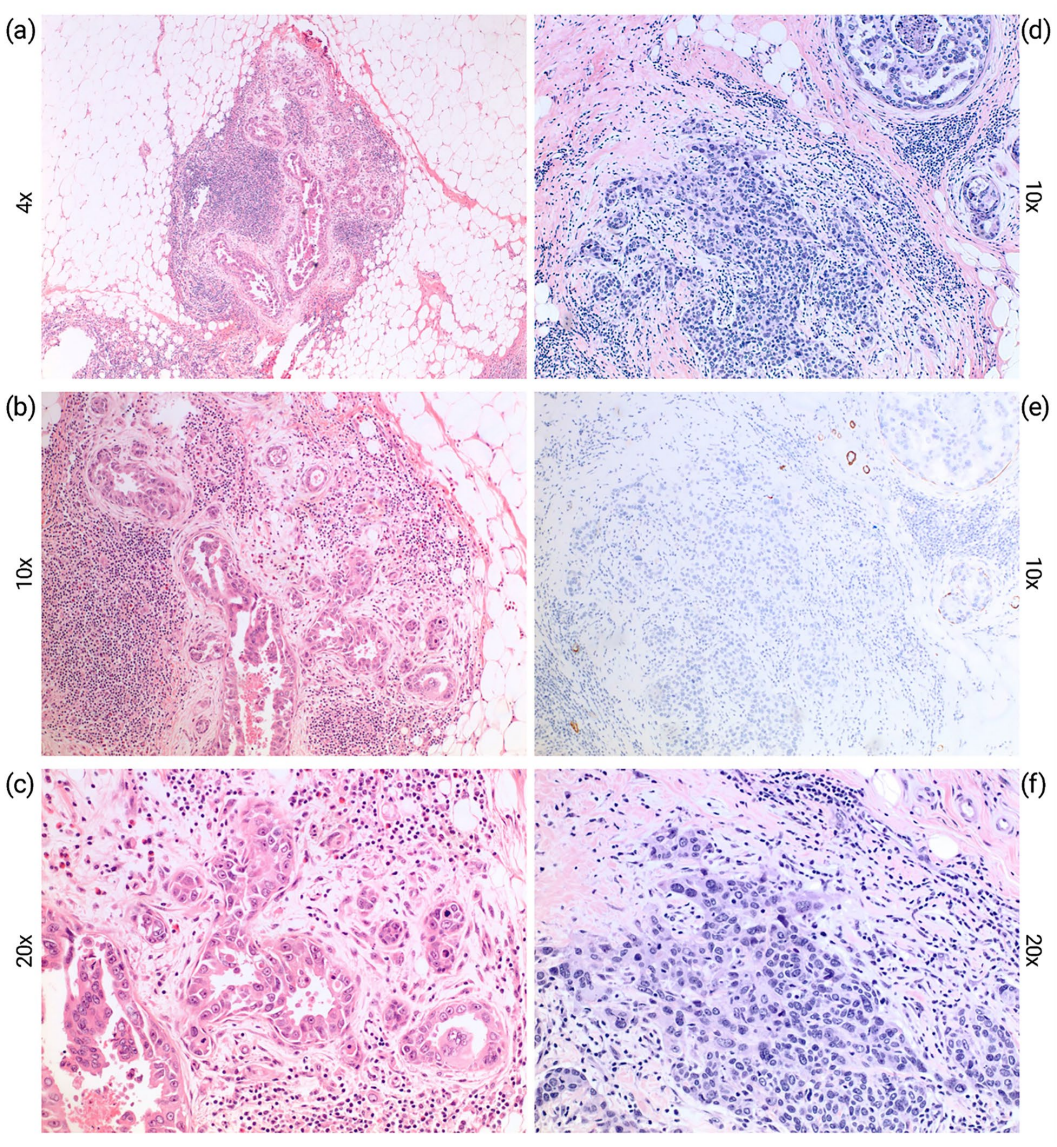
Representative photomicrographs from patients diagnosed with microinvasive carcinoma. (**a**–**c**) Microinvasive carcinoma (0.9 mm), arising in the background of ductal carcinoma in situ (DCIS) with high nuclear grade with comedo necrosis; (**d**–**f**) microinvasive carcinoma (0.8 mm) arising in ductal carcinoma in situ (DCIS), solid and cribriform patterns, with comedonecrosis and associated microcalcifications. The invasive component is negative for smooth muscle myosin heavy change by immunostaining (**e**).

All cases were reviewed by two breast pathologists. Each focus of microinvasion was measured individually, with multiple foci not being added together. Figure 1 demonstrates histological findings in MIBC, including findings seen during immunostaining. The extent of DCIS was estimated by sequential sectioning of the slices or calculated from the ratio of number of blocks with DCIS to total slides or by estimating the number of centimeters of DCIS disease present comprising the number of blocks of DCIS multiplied by 4 mm in non-sequential setting^20^. We chose a 2 mm cutoff for close margins as used by other groups^21^.

### Immunohistochemistry

Anti-estrogen receptor (ER; 1:50; Dako, Santa Clara, CA; clone 1D5), progesterone receptor (PR; 1:400; Dako; clone 1A6), HER2/neu (Dako HercepTest), anti-p63 (1:100; Biocare; clone 4A4), anti- calponin (1:500; Dako; clone CALP), and anti-smooth muscle heavy chain (1:100, Cell Marque; clone CMC569) were used for immunohistochemistry. Immunoreactivity was detected using the Dako EnVision method according to the manufacturer’s recommended protocol. Immunohistochemistry for ER and HER-2 was scored according to expression guidelines published by updated College of American Pathologists (CAP) and the American Society of Clinical Oncology (ASCO)^22^, which was recently updated in 2018^23^. ER and PR were reported as positive when greater than 1% of tumor nuclei showed staining and negative when less than 1% of tumor nuclei showed staining, HER-2 was reported as negative if scored as 1 + and positive when scored as 3 + . Tumors scored as 2 + underwent confirmatory testing with chromogenic in situ hybridization (CISH).

### Chromogenic in situ hybridization

HER-2 CISH was performed by a VENTANA 4B5 Inform HER-2 dual-color on the BenchMark Ultra system (INFORM HER2 DNA dualcolor assay—Roche Tissue Diagnostics, VENTANA Medical Systems, SA) per the manufacturer-dictated protocol. Invasive breast cancer and synchronous DCIS components were scored and recorded separately according to the updated ASCO/CAP guidelines^23^: a HER-2 copy number of 6.0 or higher per cell or a HER-2*:*CEP17 ratio of 2 or higher was considered to represent HER-2 amplification. HER-2 copy numbers of < 4 signals per cell and HER-2*/*CEP17 ratios of < 2 was nonamplified.

### Statistical analysis

The Fisher’s exact test was used to determine differences in proportions and t-tests were used to compare differences in means for parameters. All tests were 2-sided. Kaplan–Meier survival analysis was used to evaluate disease free survival (DFS) rate as a function of time, while the log-rank method was used to compare differences between groups. Univariate and multivariate analyses of DFS were performed by Cox proportional-hazard regression. For DFS variables were dichotomized as follows: patient age (**≥ **50 vs < 50), SLNB (Performed vs Not Performed), surgery type (breast conserving therapy (BCT) vs mastectomy (MST), radiation status (+ vs −), DCIS size (**≥ **25 mm vs < 25 mm), MIBC foci (≥ 2 vs 1), Margin status (+ /close vs −), DCIS nuclear grade (3 vs 1/2), pathologic necrosis (+ vs −), ER status (+ vs −), PR status (+ vs −) and HER-2 status (+ vs −). Fishers exact test and t-tests analyses were performed using SPSS statistics version 23.0.0.3. Cox-regression was performed for disease-free survival (DFS) on RStudio 2021.09.1 + 372 "Ghost Orchid". A p-value < 0.05 was considered statistically significant.

## Results

The patient characteristics and pathology findings are summarized in Table 1. The mean age of our cohort was 56.7 years. Of the 72 cases of MIBC, 50 patients received BCT/lumpectomy, and 22 underwent MST. 43 patients underwent SLNB at the time of primary surgery, while 29 did not. Sentinel lymph node metastases were identified in 2 patients, 1 had a macro metastasis (> 2 mm), and 1 had isolated tumor cells (defined as < 0.2 mm with < 200 cells). Due to the low nodal positivity rate, there were no significant associations for SLNB positivity between patient’s age, SLNB status, surgical procedure, radiation status, DCIS size, MIBC foci, margin status, nuclear grade, histologic necrosis, as well as ER, PR, and HER-2 status. Following surgery, 45 of the 72 patients received adjuvant radiation therapy (RT), while 27 patients did not.

**Table 1:** Patient characteristics and pathology findings based on treatment in microinvasive breast cancer.

	SLNB not performed	SLNB performed	P	BCT	MST	P	RT −	RT +	P
Patients (n)	29	43		50	22		27	45	
Age (years)			**0.001**			0.408			0.501
Mean size ± SEM (mm)	65.44 ± 2.44	54.58 ± 1.55		59.78 ± 1.77	57.09 ± 2.75		60.25 ± 3.05	58.17 ± 3.06	
Range (SD)	± 14.04	+ /10.19		± 12.39	± 13.73		± 15.87	± 10.28	
Surgery			**0.004**						
BCT	26	24		–	–		–	–	
MST	3	19		–	–		–	–	
Radiation status			**0.025**			**0.001**			
Positive	23	22		44	1		–	–	
Negative	6	21		6	21		–	–	
DCIS size			**0.012**			**0.001**			**0.014**
Mean ± SEM (mm)	23.72 ± 2.92	36.30 ± 3.49		25.32 ± 3.30	44.68 ± 5.31		39.03 ± 5.15	26.56 ± 2.30	
Range (SD)	± 15.73	± 22.93		± 16.28	± 24.92		± 26.79	± 15.42	
MIBC foci			0.145			0.505			0.879
Mean size ± SEM (n)	1.44 ± 0.161	2.27 ± 0.449		1.82 ± 0.290	2.22 ± 0.637		2.00 ± 0.525	1.91 ± 0.320	
Range (SD)	± 0.870	± 2.95		± 2.057	± 2.990		± 2.732	± 2.151	
Margin status			0.739			1.000			0.944
Positive	4	6		7	3		3	7	
Close	10	14		17	7		9	15	
Negative	15	23		26	12		15	23	
Nuclear grade			**0.023**			0.644			0.707
1	6	1		6	1		3	4	
2	9	12		15	6		9	12	
3	14	30		29	15		15	29	
Necrosis			0.159			0.123			0.771
Present	20	36		36	20		22	34	
Absent	9	7		14	2		5	11	
ER status			**0.044**			0.603			0.613
Positive (46)	23	23		33	13		18	28	
Negative	6	19		17	9		8	17	
PR status			**0.048**			0.756			0.383
Positive	15	10		17	8		11	14	
Negative	7	16		17	6		7	16	
HER-2 status			0.083			0.057			0.772
Positive	6	14		11	9		8	12	
Negative	16	12		23	5		10	18	

*SLNB* sentinel lymph node biopsy, *BCT* breast conservative therapy, *MST* mastectomy, *RT* radiation therapy, *SEM* standard error of the mean, *SD* standard deviation.

The bold indicated statistical significance.

Broadly, patients were placed into the following RT patient groups by surgical status: BCT with whole breast RT (37); BCT with partial breast RT (2); BCT with not otherwise specified (NOS) RT (5); BCT without RT (6); MST with post mastectomy RT (1); MST without RT (21). And into the following RT patient groups by SLNB status: SLNB not performed with whole breast RT (21); SLNB not performed with partial breast RT (1); SLNB not performed with NOS RT (1); SLNB not performed without RT (6); SLNB performed with whole breast RT (17); SLNB performed with partial breast RT (1); SLNB performed with NOS RT (4); SLNB performed without RT (21).

The mean DCIS size was 31 mm and nuclear grading was as followed: grade 1 (7), grade 2 (21), grade 3 (44). 56 of the DCIS cases had necrosis, while 16 did not. ER, PR, and HER-2 were tested for concordant positivity in both the MIBC and DCIS components. ER was positive in 46 patients and negative in 16 patients. PR was positive in 25 patients and negative in 23. HER-2 was positive in 20 patients and negative in 28 patients.

### Clinicopathological features

Many features were found to be associated with treatment/management decisions and long-term outcomes. First, SLNB was more common in patients in younger patients (P = 0.001) and in those who underwent mastectomy (P = 0.004), and RT (P = 0.025). SLNB was also more commonly used in larger DCIS size (P = 0.012), DCIS with high nuclear grade (P = 0.023), and in patients who were ER (P = 0.044) and PR (P = 0.048) positive. The use of BCT was more commonly followed with adjuvant RT when compared to patients which had mastectomy (P = 0.001) and with a larger DCIS size (P = 0.001). RT was found to be associated with less extensive DCIS (P = 0.014). The remaining clinicopathological features were not statistically significant.

### Disease free survival

DFS was defined as by the presence of recurrence or death. The average mean patient follow-up time was 55 months. Follow up for those without SLNB was 47 month, and 61 months for patients who underwent SLNB. Three patients with MIBC had recurrence and two deceased related to breast cancer mortality, leaving 5 patients in total with poor DFS and a DFS rate of 91.7%. The characteristics for these patients are presented in Table 2.

**Table 2:** Characteristics of patients with poor long-term outcomes.

Patient	Age	SLNB performed	DCIS size (mm)	MIBC foci	Margin status	Nuclear grade	Necrosis	ER	PR	HER-2	Treatment	Outcome
1	68	Yes	18	1	Close	3	Present	Neg	Neg	Pos	BCT, RT(+)	Death (45 months)
2	40	Yes	40	1	Neg	3	Present	Pos	–	–	MST, RT(−)	Recurrence (113 months)
3	78	No	50	1	Neg	3	Present	Pos	–	–	MST, RT(−)	Death (20 months)
4	98	No	68	3	Pos	2	Present	Pos	Neg	Neg	BCT, RT(−)	Recurrence (21 months)
5	52	Yes	24	1	Close	3	Present	Pos	Pos	Neg	BCT, RT(−)	Recurrence (12 months)

*SLNB* sentinel lymph node biopsy, *DCIS* ductal carcinoma in situ, *MIBC* microinvasive breast cancer.

None of the following variables were significant following univariate or multivariate cox regression for DFS (P > 0.05). Results for Cox-regression can be found in Supplemental Table S1, while Fisher’s exact and t-test results can be found in Supplemental Table S2.

## Discussion

The present work evaluated clinical parameters including SLNB, surgical type, radiation status, and long-term outcomes in relation to histological features in MIBC and associated DCIS. Overall, MIBC was found to have a favorable prognosis and a DFS rate of 93.1%, congruent with other studies which have shown recurrence free survival rates between 90 and 97%^6,9,10,24^. These studies had an average follow up time of 64.9 months, an overview of all MIBC publications based on the 1 mm AJCC cutoff is summarized in Table 3.

**Table 3:** Significant literature based on the 1 mm AJCC categorization of MIBC.

Study	Institution	Years	Case number	SLNB (%)	BCT (%)	RT %)	Follow up (months)	Survival
Present study	Brown	2002–2021	72	43 (60%)	50 (69%)	45 (63%)	55 (mean)	DFS 93.1%
Zhang, 2021^16^	University of Rochester	2007–2019	46	33 (72%)	21 (46%)	15 (33%)	38 (median)	RFS 100%
Si/2020^26^	Jiaxing University	2006–2015	359	242 (67%)	26 (7%)	17 (5%)	61 (median)	OS 99.36%
Zhang, 2020^31^	Hebei Medical University	2011–2018	264	164 (62%)	23 (9%)	19 (72%)	47 (median)	RFS 95.4%
Kim, 2018^12^	Seoul National University	2003–2014	136	110 (81%)	55 (40%)	–	48 (median)	RFS 97.8%
Pu, 2018^32^	Sichuan University	1997–2014	242	–	26 (11%)	23 (9%)	109 (median)	DFS 96.89%
Li, 2015^33^	Tianjin Medical University	2003–2009	93	2 (2%)	1 (1%)	–	100 (median)	RFS 90.9%
Wang, 2015^17^	Tianjin Medical University	2002–2009	131	–	–	–	69 (median)	DFS 95.2%
Matsen, 2014^27^	Memorial Sloan Kettering Cancer Center	1997–2010	414	414 (100%)	198 (48%)	174 (42%)	59 (median)	RFS 95.9%
Shatat, 2013^34^	University of Kansas	1998–2012	40	–	19 (48%)	–	30 (mean)	OS 100%
Kapoor, 2013^35^	John Wayne Cancer Institute	1995–2010	45	31 (69%)	24 (53%)	–	83 (median)	RFS 93.7%
Margalit, 2012^6^	Harvard	1997–2005	83	53 (64%)	52 (63%)	53 (64%)	77 (median)	RFS 94.7%
Lyons, 2012^36^	Memorial Sloan Kettering cancer center	1996–2004	112	111 (100%)	60 (53%)	51 (46%)	72 (median)	DFS 91%
Pimiento, 2011^37^	H.Lee Moffitt cancer center	1996–2009	87	87 (100%)	59 (68%)	0 (0%)	74 (median)	OS 94.2%
Parikh, 2010^10^	Yale	1973–2004	72	4 (6%)	72 (100%)	72 (100%)	107 (median)	RFS 90.7%
Vieira, 2010^22^	New York University	1993–2006	21	14 (67%)	(55%)	–	36 (mean)	OS 100%
Kwon, 2010^9^	Seoul National University	2000–2006	120	–	(53%)	30 (25%)	61 (median)	RFS 97.2%

*AJCC* American Joint Committee on Cancer, *SLNB* sentinel lymph node biopsy, *BCT* breast conservative therapy, *RT* radiation therapy, *DFS* disease free survival, *RFS* recurrence free survival, *OS* overall survival.

In the present study, the use of SLNB was more often confined to patients undergoing mastectomy and was also performed more commonly in the background of DCIS showing high nuclear grades with ER negative and HER-2 positive receptor status. Only 1 of 44 patients (2.3%) had positive lymph nodal metastasis (> 0.2 mm) following SLNB and no variable was able to statistically predict nodal positivity. A finding possibly secondary to the low propensity of lymph node metastasis in MIBC. Importantly, when comparing patients who had undergone SLNB to those that had not, there was no difference in long term outcome in our study. Although 4 out of 5 patients (80%) who experienced poor DFS outcomes did not receive radiotherapy.

Despite the lack of differences in long term outcome following SLNB, surgical complications associated with SLNB biopsy have been reported. In a large study of 5327 patients performed by Wilke et al.^25^, complications included axillary wound infection (1.0%), axillary seroma (7.1%), and axillary hematoma (1.4%). For older patients, SLNB was also found to be associated with an increased incidence of axillary seroma.

We also observed HER-2 positivity to be relatively common in MIBC, and when compared with pure DCIS, numerous studies have reported a higher rate of HER-2 overexpression in MIBC than in both invasive carcinomas and DCIS^6,12^. This is counter intuitive, as HER-2 amplification has been traditionally seen to occur more often in DCIS than in invasive carcinoma^26^. Zhang et al.^16^ demonstrated HER-2 positivity to be associated with high-grade morphologic features, but not nodal metastasis or worse outcomes. We also did not find HER-2 overexpression to be associated with recurrence in MIBC, despite the fact that HER-2 has been demonstrated to be an independent high risk predictor of early recurrence in invasive breast cancer^27^. These findings could be secondary to good overall outcomes in MIBC, as well as the small patient population in our study. Nonetheless, the role of HER-2 targeted therapy in MIBC will need to be validated in the clinical setting by future studies.

In the present study we analyzed clinicopathological parameters such as the number of microinvasive foci, and both the extent and histology of background DCIS, however none of these were found to be associated with long term clinical outcomes. Although some studies have found patients with multiple foci of microinvasion to have worse DFS outcomes^28^, a large study of 414 patients performed by Matsen et al.^29^ showed that there was no higher risk of nodal involvement for patients with ≥ 2 foci of microinvasion when compared to 1 focus.

In a recent study from the Surveillance Epidemiology and End Results (SEER) database between 2003 and 2015, Chen et al.^30^ examined nodal metastasis, axillary surgery, and prognosis in 11,692 MIBC patients. Multivariate analyses showed that nodal metastasis was the best survival predictor; however, axillary lymph node dissection did not demonstrate a statistically significant survival benefit at 10-years.

There were several pitfalls in the present study. This was a retrospective study which always carries the risk of selection bias and the follow up time was relatively short. We also did not perform molecular testing and due to the low incidence of MIBC we were unable to increase our cohort size. Multi-institution collaboration will be important for future studies.

In summary, when comparing patients who had undergone SLNB to those who had not we found no difference in long term clinical outcomes. The decision to undergo SLNB in MIBC should be made with the knowledge that surgical complications are reported, and traditional metrics for risk stratification and treatment decision making in invasive mammary carcinoma may not hold true in the setting of microinvasion.

## Supplementary Information

Supplementary Table 1.

Supplementary Table 2.

## Data Availability

The datasets used and analyzed during the current study are available from the corresponding author upon reasonable request.
