# Supplementary material for: Microinvasive breast cancer and the role of sentinel lymph node biopsy
Source: Sci Rep. 2022 Jul 20;12:12391. doi: 10.1038/s41598-022-16521-8 (PMC9300703; doi:10.1038/s41598-022-16521-8)
Supplement: Supplementary file 1 — Supplementary Table 1. [file 41598_2022_16521_MOESM1_ESM.docx]

|  | **Univariate HR** | **Confidence Interval** | **P Value** | **Multivariate**  **HR** | **Confidence Interval** | **P Value** |
| --- | --- | --- | --- | --- | --- | --- |
| Age (years)  ≥50 vs <50 | 1.44 | 0.1487- 13.95 | 0.753 | 7.212e+07 | 0-Inf | 1.000 |
| SLNB  Performed vs Not Performed | 0.899 | 0.149- 5.43 | 0.908 | 1.235e+05 | 0-Inf | 0.999 |
| Surgery  BCT vs MST | 0.817 | 0.114-  5.851 | 0.841 | 1.738e-29 | 0-Inf | 0.999 |
| Radiation status  + vs - | 0.1911 | 0.020-  1.800 | 0.148 | 3.496e-21 | 0-Inf | 0.998 |
| DCIS size  ≥25mm vs <25mm | 1.675 | 0.280- 10.04 | 0.502 | 3.955 | 0-Inf | 1.000 |
| MIBC foci  ≥2 vs 1 | 0.2716 | 0.030- 2.434 | 0.244 | 1.055e-03 | 0-Inf | 1.000 |
| Margin status  +/close vs - | 1.566 | 0.262- 9.374 | 0.623 | 9.137e+19 | 0-Inf | 0.998 |
| Nuclear grade  1/2 vs 3 | 2.115 | 0.231-  19.41 | 0.448 | 9.910e | 0-Inf | 1.000 |
| Necrosis  + vs - | inf | 0-  inf | 0.999 | 2.813e+05 | 0-Inf | 1.000 |
| ER status  + vs - | 2.671 | 0.295- 24.12 | 0.382 | 1.822e+12 | 0-Inf | 0.999 |
| PR status  + vs - | 0.456 | 0.041- 5.043 | 0.522 | 1.077 | 0-Inf | 1.000 |
| HER-2 status  + vs - | 0.503 | 0.044- 5.639 | 0.578 | 3.296e+14 | 0-Inf | 0.998 |

**Supplemental Table 1. Univariate and multivariate analyses of long-term outcomes using the Cox Proportional-Hazard Regression. HR, Hazard Ratio; SLNB, Sentinel Lymph Node Biopsy; DCIS, Ductal Carcinoma in Situ. Signiﬁcant features (P≤0.05) are shown in bold.**
