# Supplementary material for: Microinvasive breast cancer and the role of sentinel lymph node biopsy
Source: Sci Rep. 2022 Jul 20;12:12391. doi: 10.1038/s41598-022-16521-8 (PMC9300703; doi:10.1038/s41598-022-16521-8)
Supplement: Supplementary file 2 — Supplementary Table 2. [file 41598_2022_16521_MOESM2_ESM.docx]

|  | **Good DFS** | **Poor DFS** | **P** |
| --- | --- | --- | --- |
| Patients (n) | 67 | 5 |  |
| Age (years) |  |  | 0.131 |
| Mean size  +/- SEM (mm) | 58.34+/-  1.41 | 67.20+/-  10.09 |  |
| Range (SD) | +/-11.59 | +/-22.57 |  |
| SLNB |  |  | 1.000 |
| Not performed | 27 | 2 |  |
| Performed | 40 | 3 |  |
| Surgery |  |  | 0.638 |
| BCT | 47 | 3 |  |
| MST | 20 | 2 |  |
| Radiation status |  |  | 0.062 |
| Positive | 44 | 1 |  |
| Negative | 23 | 4 |  |
| DCIS size |  |  | 0.340 |
| Mean size  +/- SEM (mm) | 30.58+/-  2.59 | 40.00+/-  9.01 |  |
| Range (SD) | +/-21.23 | +/-20.14 |  |
| MIBC foci |  |  | 0.597 |
| Mean  +/- SEM (n) | 1.99+/-  0.298 | 1.40+/-  0.400 |  |
| Range (SD) | +/-2.44 | +/-0.894 |  |
| Margin status |  |  | 0.689 |
| Positive | 9 | 1 |  |
| Close | 22 | 2 |  |
| Negative | 36 | 2 |  |
| Nuclear grade |  |  | 1.000 |
| 1 | 7 | 0 |  |
| 2 | 20 | 1 |  |
| 3 | 40 | 4 |  |
| Necrosis |  |  | 0.580 |
| Present | 51 | 5 |  |
| Absent | 16 | 0 |  |
| ER status |  |  | 0.650 |
| Positive | 42 | 4 |  |
| Negative | 24 | 1 |  |
| PR status |  |  | 0.601 |
| Positive | 24 | 1 |  |
| Negative | 21 | 2 |  |
| HER-2 status |  |  | 1.000 |
| Positive | 19 | 1 |  |
| Negative | 26 | 2 |  |

**Supplemental Table 2. Patient characteristics and pathology findings based long-term outcomes in microinvasive breast cancer. SLNB, Sentinel Lymph Node Biopsy; BCT, Breast conservative therapy; MST, Mastectomy; RT, Radiation Therapy; SEM, Standard error of the Mean; SD, Standard Deviation.**
